# Serous borderline tumor progressing to low-grade serous carcinoma after 35 years: a case of extended latency with molecular evidence of clonal relationship

**DOI:** 10.1016/j.gore.2026.102194

**Published:** 2026-08-20

**Authors:** Emily A. Towery, Elizabeth H. Stover, David L. Kolin, Rebecca L. Porter

**Affiliations:** a Department of Pathology, Brigham and Women's Hospital, Boston, MA, United States; b Department of Medical Oncology, Dana Farber Cancer Institute, Boston, MA, United States

**Keywords:** Low grade serous carcinoma, Ovarian serous borderline tumor, BRAF

## Abstract

**Background:**

Low-grade serous ovarian carcinoma (LGSOC) is an uncommon epithelial malignancy that typically arises in the setting of a serous borderline ovarian tumor (SBOT), with the majority of low-grade serous neoplasms harboring mutually exclusive mutations in *KRAS*, *BRAF*, or *NRAS*. The time to progression from SBOT to LGSOC is highly variable with a median of 10 years, with the longest previously reported interval of 39 years. LGSOC often presents at advanced stage with a high propensity for recurrence despite surgical intervention (the primary treatment modality) and systemic pharmacologic treatments.

**Case Presentation:**

Herein, we present the case of a 62-year-old patient with a diagnosis of advanced LGSOC that presented 39 years after her initial diagnosis and 35 years after last documented surgical resection of SBOT. Additionally, we describe the utilization of molecular testing for a *BRAF*^V600E^ mutation in both the SBOT and LGSOC specimens, suggesting a clonal relationship between the two neoplasms in this case, and also providing an indication for targeted therapy to which this patient demonstrated a rapid and robust response.

**Conclusion:**

This case exemplifies the long-term natural history of low-grade serous neoplasia and highlights how the incorporation of molecular studies can improve diagnostic certainty and guide therapy selection for patients. Further, we provide a review of the literature on SBOT progression to LGSOC, including risk factors for progression and therapeutic options to treat recurrent/progressive disease.

## Highlights

• Low grade serous carcinoma can arise decades following a diagnosis of ovarian serous borderline tumor.
• A multimodal approach to pathologic diagnosis (morphology, immunohistochemistry, molecular) can be crucial to deciding best clinical management.
• Identifying molecular drivers in low grade serous carcinoma can lead to additional treatment options when first line systemic therapiesfail.

## 1 Introduction

Low-grade serous ovarian carcinoma (LGSOC) is an uncommon epithelial malignancy of the ovary representing less than 10% of epithelial ovarian cancers (Prat et al., 2018). LGSOC is characterized by young age and often presents at advanced stage. Although associated with a prolonged overall survival compared with the more common high grade serous ovarian carcinoma (HGSOC), LGSOC are relatively resistant to platinum-based chemotherapy and have a high propensity for recurrence despite surgical and pharmacologic intervention (Moujaber et al., 2022; Grisham, 2016; Goulding et al., 2020).

LGSOC is thought to arise from serous adenofibroma or cystadenoma which progresses to a serous borderline ovarian tumor (SBOT) and then invasive carcinoma. The majority of low-grade serous neoplasia harbors mutually exclusive mutations in *KRAS*, *BRAF*, or *NRAS*, causing constitutive activation of the mitogen-activated protein kinase (MAPK) pathway, while *TP53* mutations are uncommon (Moujaber et al., 2022; Vang et al., 2009; Singer et al., 2005; Stover et al., 2025). Approximately 4% of SBOT will progress to LGSOC, and adverse features associated with progression to LGSOC include micropapillary architecture and extra-ovarian implants (Vang et al., 2017). The time to progression is highly variable; in a large series of 942 SBOT with long term follow up, the median time to the development of carcinoma was 10 years, with the longest interval of 25 years (Vang et al., 2017). Chui et al. reported a similar median time from progression of SBOT to LGSOC of 8.1 years (*n* = 39) with a maximum of 24.9 years (Chui et al., 2019). Silva et al. reported a case with an interval of 39 years between diagnosis of stage 1 SBOT and recurrence as LGSOC, highlighting the potential for a decades-long interval between SBOT and LGSOC (Silva et al., 1998).

Herein, we present a case of LGSOC that presented 39 years after the initial diagnosis of the SBOT, with an interval of 35 years from last surgical debulking of SBOT until subsequent progression to LGSOC, and provide molecular evidence suggesting a clonal relationship between the two neoplasms. This case exemplifies the long-term natural history of low-grade serous neoplasia and highlights how the incorporation of molecular studies can improve diagnostic certainty and potentially guide therapy selection.

## 2 Case description

A 62-year-old with a remote history of recurrent serous borderline tumors (Table 1) presented with pulmonary masses, skin nodules, and adenopathy on imaging concerning for metastatic carcinoma. She had initially presented at the age of 23 with a pelvic mass for which she underwent a total abdominal hysterectomy with bilateral salpingo-oophorectomy and omentectomy with pathology demonstrating stage III SBOT involving both ovaries and with non-invasive implants involving the appendix and omentum. The patient did not receive adjuvant systemic therapy and underwent additional laparoscopies with debulking of recurrent peritoneal non-invasive implants over the following 4 years. She then continued on surveillance for a number of years.

**Table 1:** Timeline of Diagnosis and Treatment History.

Year	Month	Summary of events
1983	May	Initial surgery (total hysterectomy and bilateral oophorectomy) with pathology revealing SBOT
1983–1987		Multiple debulking surgeries for recurrent SBOT
1988–2022		Patient discontinued contact with medical system and focused on diet and exercise
2022	May	Presented with Horner syndrome; imaging reveals widespread metastatic disease; CA125 = 327 U/mL
	June–July	Biopsies of supraclavicular lymph node and abdominal masses show metastatic low grade serous carcinoma; sequencing from lymph node metastasis identifies *BRAF*^V600E^
	August	Initiated on carboplatin and paclitaxel
	December	PET-CT shows partial response; letrozole added and later switched to anastrozole
2023	January	Completed 6 cycles of carboplatin and paclitaxel; ddPCR identifies *BRAF*^V600E^ on samples from 1987 surgery
	February	PET-CT shows stable disease; CA125 = 174 U/mL
	March	Patient discontinued anastrozole; patient wishes to focus on diet
	March–June	Discussion of possible treatments including targeted therapies; patient declines
	December	CT scans show stable disease; patient wishes to avoid any additional treatment as long as possible; CA125 = 166 U/mL
2025	December	CT shows interval increase in metastatic disease burden, as evidenced by increase in size of multiple soft tissue masses and of a peritoneal nodule
2026	January	Biopsy of right flank soft tissue mass confirmed metastatic low grade serous ovarian carcinoma, positive for BRAF^V600E^ immunohistochemistry
	February	CA125 = 488 U/mL. Initiated dabrafenib 75 mg BID
	March	CT scans after 6 weeks show excellent treatment response with decrease in all areas of metastatic disease
	April	CA125 = 63 U/mL. Trametinib 1 mg daily added to dabrafenib

**Fig. 1:**
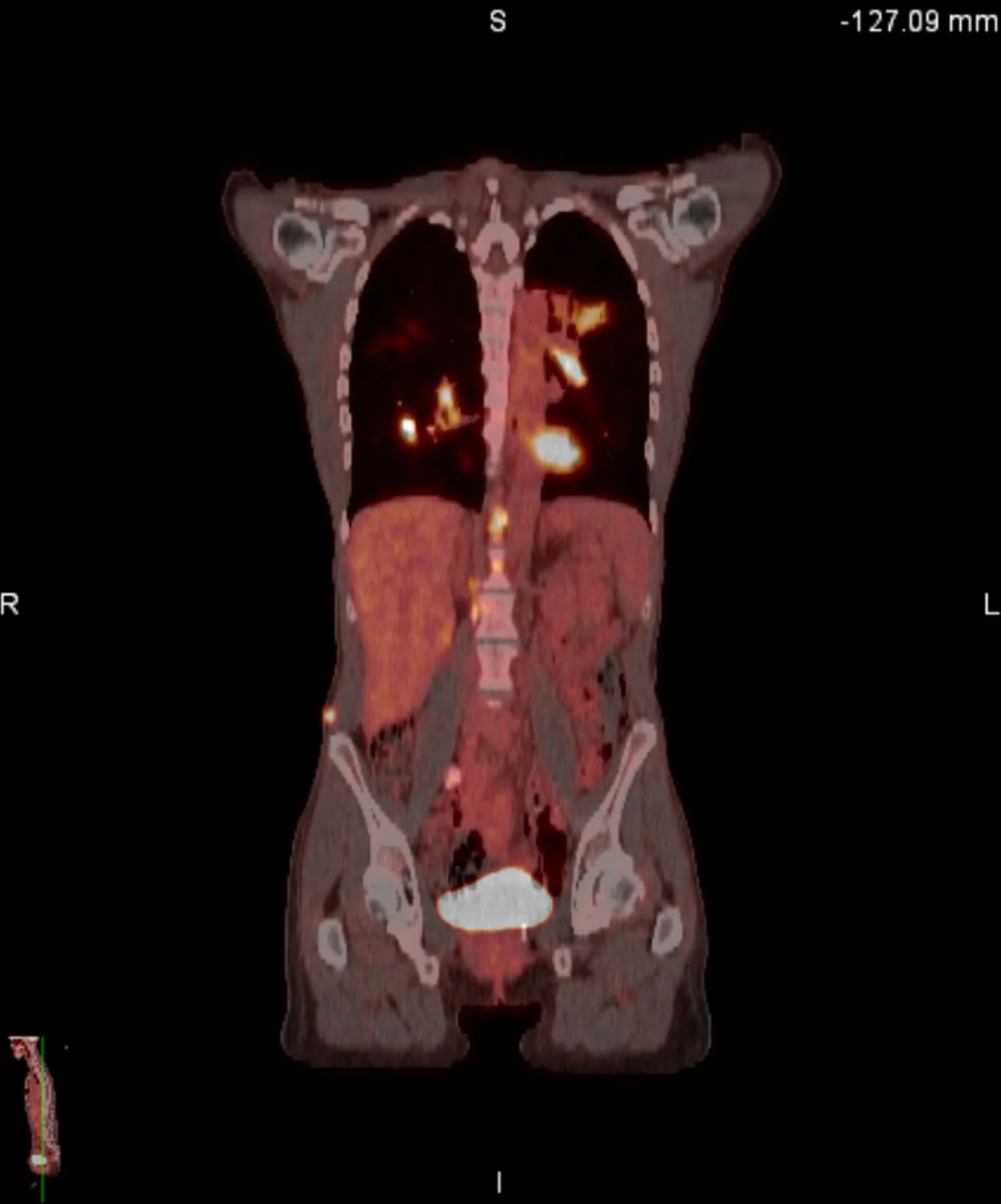
Whole body PET-CT showing widespread metastases to lung, lymph nodes, and skin.

**Fig. 2:**
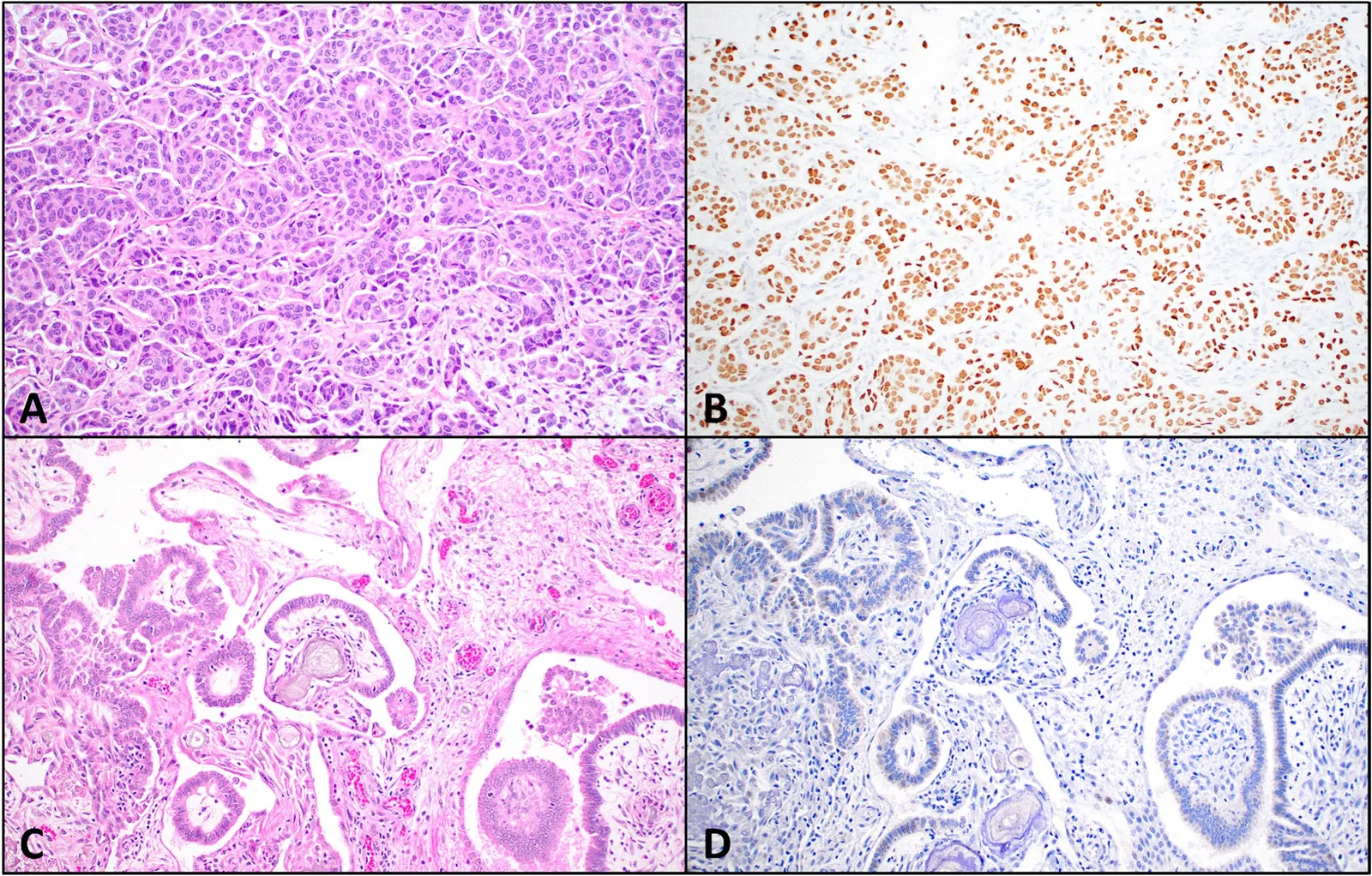
**A-B.** Metastatic low grade serous carcinoma. A. H&E morphology (20×). B. PAX8 immunohistochemistry (20×). C—D. Serous borderline ovarian tumor. C. H&E morphology (20×). D. BRAFV600E immunohistochemistry (20×).

The patient was subsequently lost to follow up until the age of 62, 39 years following the initial diagnosis, when she re-presented with Horner syndrome. Imaging revealed a suprahilar, paramediastinal/left upper lobe lung mass abutting the aorta (9 cm in greatest dimension), innumerable pulmonary nodules, some of which were calcified, lymphadenopathy above and below the diaphragm, and multiple FDG-avid cutaneous and subcutaneous nodules (Fig. 1). CA125 was 327 U/mL. The patient underwent excision of a supraclavicular lymph node with pathology showing metastatic carcinoma of unknown primary. An initial immunohistochemical panel demonstrated the tumor was positive for AE1/AE3 and CK7, and negative for CK20, CDX2, CD56, chromogranin, synaptophysin, TTF-1, and napsin A. Additional material was not available for additional immunohistochemistry, and the limited immunoprofile was not specific for a site of origin. Tissue was sent for targeted panel next generation sequencing in the face of a carcinoma of unknown primary, which revealed a low tumor mutational burden (1 mutation/Mb) and identified *BRAF*^V600E^ and *ARID1A* L159fs + 21 pathogenic mutations. The patient underwent additional sampling of a subcutaneous soft tissue nodule of the right pelvis/abdomen which demonstrated metastatic LGSOC (Fig. 2A). Immunohistochemistry performed on this mass showed positive staining for PAX8 (Fig. 2B), WT-1, CK7, ER (90%, strong), PR (10%, weak), with wild-type staining for p53, a profile consistent with LGSOC. Immunohistochemistry for mismatch repair proteins MLH1, MSH2, MSH6, and PMS2 revealed intact expression, and HER2 immunohistochemistry was 2+ (gastric criteria). Select sections of the initial serous borderline tumors were available for re-review, which showed usual type SBOT architecture lacking micropapillary/cribriform features (Fig. 2C). A PAX8 immunostain, performed at the time of re-review of the initial borderline tumor, showed positive staining.

To investigate the potential relationship between the current metastatic LGSOC and prior SBOT, a *BRAF*^V600E^-specific immunohistochemical stain was performed on archival material from one of the debulking surgeries performed 4 years following her initial diagnosis and 35 years prior to her diagnosis of LGSOC. The stain was interpreted as equivocal, possibly owed to the age of the formalin-fixed paraffin-embedded tissue (Fig. 2D). The same 35-year-old material was then sent for *BRAF* digital droplet polymerase chain reaction (ddPCR) which detected the presence of a *BRAF* c. 1799 T > A (V600E) variant at a fractional abundance of 9% (271 mutant events and 2651 wild-type events).

Upon diagnosis of metastatic LGSOC, the patient was initiated on standard of care carboplatin and paclitaxel. The treatment course was complicated by neurocysticercosis following cycle 2, which was treated with albendazole and dexamethasone during which time chemotherapy was held and then eventually resumed. Following cycle 4 of carboplatin and paclitaxel, interval PET-CT revealed a partial response to therapy with decreased FDG avidity in cutaneous and subcutaneous lesions as well as some of the pulmonary nodules with others remaining stable. Letrozole was added to chemotherapy with cycle 5 and was subsequently switched to anastrozole due to adverse effects. Following the 6th cycle of carboplatin and paclitaxel with anastrozole, interval CT scans obtained revealed numerous stable bilateral calcified and noncalcified cutaneous and subcutaneous nodules, calcified mediastinal and retroperitoneal adenopathy and no new sites of disease. A CA125 at this time was 174 U/mL, and the patient opted to discontinue anastrozole and chemotherapy given personal preferences. She sought several additional medical opinions with providers who discussed therapies targeting the known *BRAF*^V600E^ mutation as well as hormonal therapies and/or antiangiogenic therapies. The patient opted to pursue alternative therapies. Interval restaging CT scans obtained 9 months off therapy revealed overall stable disease with CA125 166. She briefly resumed aromatase inhibitor therapy with letrozole, but self-discontinued it after several days for personal reasons.

**Fig. 3:**
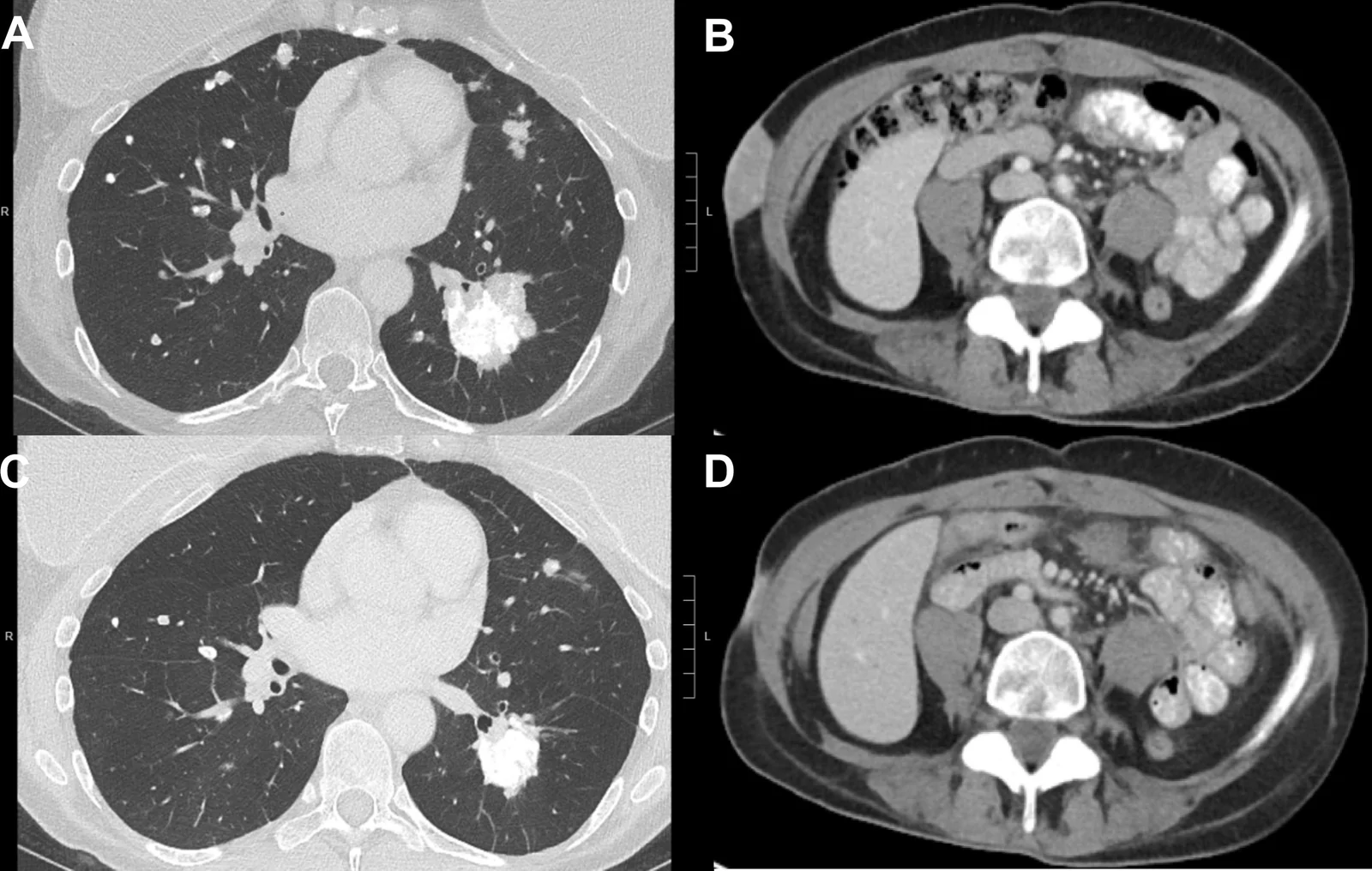
A-B: CT scans prior to initiation of dabrafenib monotherapy highlighting multiple pulmonary nodules (A) and a R flank soft tissue mass (B). C-D: CT scans obtained after 6 weeks of dabrafenib monotherapy demonstrating significantly reduced size of multiple pulmonary nodules (C) and R flank soft tissue mass (D).

The patient was again lost to follow up until she re-presented to Medical Oncology reporting increasing multifocal pain, cough and shortness of breath, and growth of her prior cutaneous lesions. On exam she was noted to have multiple cutaneous soft tissue masses and laboratory evaluation was notable for CA125 of 413. CT scans of the chest, abdomen, pelvis and neck were obtained which revealed an overall interval increase in metastatic disease burden, with increased size of bilateral pulmonary metastases (Fig. 3A), left neck and chest wall cutaneous/subcutaneous nodules, multiple soft tissue masses, a peritoneal nodule in the abdomen and pelvis with the largest in the right flank (Fig. 3B), and new left supraclavicular soft tissue mass. She subsequently underwent an image-guided biopsy of a superficial right flank soft tissue mass which confirmed metastatic LGSOC, positive by immunohistochemistry for BRAF^V600E^, ER (moderate to strong, 40%) and HER2 2+ (gastric criteria). At this time, CA125 was 488 and the patient was agreeable to treatment and initiated single agent dabrafenib 75 mg BID with plan to add trametinib once tolerance was established. After 17 days on dabrafenib monotherapy, the patient was seen in the office and exam was notable for a marked decrease in her cutaneous lesions. At six weeks on therapy, repeat CT scans revealed a decrease in the size of multiple pulmonary nodules (Fig. 3C), left axillary and left suprahilar lymph nodes, cutaneous/subcutaneous soft tissue nodules (Fig. 3D), abdominal wall metastases, and a retroperitoneal nodule. CA125 at week 8 of therapy was 63. At this time, trametinib 1 mg daily was added to dabrafenib, and patient continues on dual therapy at last follow up.

## 3 Discussion

LGSOC and HGSOC are now well-established as distinct clinicopathologic entities, with differences in morphology, molecular alterations and drivers, and clinical behavior. LGSOC remains less well-represented in medical literature and clinical trials in part due to comprising a minority of ovarian serous cancers (<10%) and only 5–8% of all ovarian carcinomas (Goulding et al., 2020; Köbel et al., 2010). In the majority (>75%) of LGSOC, precursor lesions are identified concurrently with invasive tumor, and studies have shown no difference in clinical behavior of de novo stage II-IV LGSOC and LGSOC precursor lesions that recur as LGSOC (Vang et al., 2009; Shvartsman et al., 2007; Ahn and Longacre, 2016).

Specific features that influence the risk of progression from SBOT to LGSOC remain incompletely understood. Histologic features associated with progression to LGSOC include presence of implants and whether or not they are invasive (non-invasive implants hazard ratio [HR] = 7.7; invasive implants HR = 42.3) and micropapillary/cribriform growth pattern (HR = 3.8) (Vang et al., 2017). Given the high risk of progression, current WHO classification considers invasive implants as equivalent to LGSOC (Vang et al., 2020). Recent advances in the molecular characterization of LGSOC reveal the presence of *KRAS* mutations in 33%, *BRAF* mutations in 8–11%, and *NRAS* mutations in 11% (Manning-Geist et al., 2022; Gershenson et al., 2022a). *BRAF* mutations are more prevalent in SBOTs (23–48%) than LGSOC, suggesting that SBOTs with *BRAF* may be less likely to progress (Moujaber et al., 2022; Goulding et al., 2020; Wong et al., 2010; Turashvili et al., 2018). In a series of 201 SBOTs followed longitudinally, the presence of *BRAF* mutations was associated with a lower risk of subsequent serous carcinoma compared with tumors with *KRAS* or neither mutation (Chui et al., 2021). Conversely, among 39 cases of advanced SBOTs, the presence of *KRAS* mutations was associated with higher risk of recurrence (McHenry et al., 2023). Within LGSOC, tumors lacking mutations in the MAPK pathway have a worse prognosis than those harboring *BRAF*, *KRAS*, or *NRAS* mutations (Manning-Geist et al., 2022; Gershenson et al., 2022a).

In this case, despite the extremely long interval between last SBOT therapy and diagnosis of LGSOC, the fact that both the remote non-invasive implant and the subsequent disseminated LGSOC harbored a *BRAF*^V600E^ mutation supports the SBOT as being a precursor to the LGSOC. Additionally, given the typical indolent growth pattern of LGSOC, it is possible that the recurrence was initiated significantly earlier than it came to clinical attention. However, the possibility that the LGSOC arose de novo from a separate process (e.g., peritoneal endosalpingiosis) and acquired the same mutation cannot be definitively excluded. This case also highlights the potential utility of ddPCR in select cases where analysis of a known gene mutation is warranted. Panel-based targeted NGS is routinely used clinically to molecularly profile tumors. However, in our experience, it has limited success on very old formalin-fixed paraffin-embedded tissue. In contrast, ddPCR is a highly sensitive, specific, and cost-effective technique which successfully identified the *BRAF* mutation in nearly 40-year-old tissue (Olmedillas-López et al., 2017). ddPCR is more often utilized in liquid biopsy specimens, though targeted use in FFPE samples to identify a variety of molecular alterations, including *BRAF*^V600E^, has been employed in various tumor types (Heredia et al., 2013; O'Hern et al., 2023; Sharma et al., 2025). The ddPCR methodology is, however, limited in comparison to NGS as it is a single gene assay, and is best suited to interrogate for the presence of a specific mutation (Olmedillas-López et al., 2017).

Surgery is the primary treatment modality for the management of SBOT and LGSOC; residual disease following primary cytoreduction remains the strongest predictor of prolonged survival, and studies have shown secondary cytoreductive surgery for recurrent disease to be of benefit to select patients (Goulding et al., 2020; Crane et al., 2015). Platinum-based chemotherapy remains the standard first line systemic therapy option, but LGSOC is relatively chemoresistant in comparison to HGSOC and alternative systemic therapies including anti-angiogenic, hormonal and targeted therapy combinations are being investigated (Zwimpfer et al., 2023; Gershenson et al., 2009; Grabowski et al., 2016; Grisham et al., 2023; Musacchio et al., 2025). Retrospective data has demonstrated benefit of hormonal therapies such as aromatase inhibitors and tamoxifen as maintenance following first line chemotherapy and also as treatment for recurrent disease (Gershenson et al., 2017; Gershenson et al., 2012). The efficacy of hormonal therapy without chemotherapy in the first line was tested prospectively in the NRG-GY019 trial (NCT04095364). However, the trial did not demonstrate noninferiority of letrozole compared to chemotherapy and letrozole in the overall cohort, meeting the prespecified criteria for early termination for futility (Fader et al., 2026); thus, chemotherapy remains the standard of care first line therapy for most patients with advanced LGSOC.

The management of recurrent LGSOC following prior chemotherapy has been rapidly evolving (Table 2). Based on the similarities of hormonal dependence and resistance mechanisms to hormonal therapies between LGSOC and hormone receptor-positive breast cancers, cyclin-dependent kinase 4/6 (CDK4/6) inhibitors in combination with hormonal agents have been found to have efficacy in LGSOC (Cobb et al., 2022; Slomovitz et al., 2026). The prevalence of genomic aberrations resulting in activation of the MAPK pathway in LGSOC has also led to the investigation of MEK and BRAF inhibitors as potential therapeutic options for patients with recurrent LGSOC, including the phase III GOG-0281 which showed improved PFS and ORR with trametinib compared with standard of care therapies (Stover et al., 2025; Gershenson et al., 2022b). More recently, the combination of avutometinib, a dual RAF/MEK clamp that inhibits both MEK activity and RAF-mediated MEK phosphorylation, and the FAK inhibitor, defactinib, has received accelerated United States Food and Drug Administration (FDA) approval for patients with recurrent LGSOC harboring *KRAS* mutations who have received prior systemic therapy (Blair, 2025). This approval was based on the phase 2 RAMP 201 trial (Banerjee et al., 2025) and the confirmatory randomized phase 3 RAMP 301 is ongoing (NCT06072781) (Grisham et al., 2025). For patients with *BRAF*^V600E^-mutated tumors, there is biologic rationale for the use of BRAF inhibitors as monotherapy or in combination with MEK inhibitors based largely on data from patients with *BRAF*^V600E^-mutated melanoma patients (Zhou and Johnson, 2018; Robert et al., 2019; Dummer et al., 2018). While there is limited data specifically in patients with LGSOC, the combination of the BRAF inhibitor, dabrafenib, and the MEK inhibitor, trametinib, has accelerated tumor-agnostic approval for patients with *BRAF*^V600E^-mutated solid tumors (Salama et al., 2020). Additionally, durable responses to BRAF inhibitor monotherapy have also been reported in small numbers of patients with LGSOC (Moujaber et al., 2022; Combe et al., 2015; Hyman et al., 2015). Ongoing clinical trials evaluating novel MEK inhibitor combinations, mutant-specific KRAS inhibitors (e.g. KRAS G12C or G12D inhibitors) and pan-RAS inhibitors, and targeted therapies in combination with hormonal therapies to overcome resistance may lead to new therapeutic options for patients with LGSOC (Stover et al., 2025; Zwimpfer et al., 2023).

**Table 2:** Selected Targeted and Endocrine Therapies for Advanced LGSOC.

Agent	Patient Population and Regimen	Trials	Relevant Trial Findings
Letrozole	Letrozole +/− Carboplatin and Paclitaxel in stage II-IV newly diagnosed LGSOC	NRG-GY019 (NCT04095364, phase III) (Fader et al., 2026)	Letrozole monotherapy did not demonstrate noninferiority compared to chemotherapy + letrozole
Anastrozole	Anastrozole monotherapy in recurrent, ER-positive LGSOC	PARAGON (phase II) (Tang et al., 2019)	CBR at 3 months of 64% (95% CI 48%–78%); median duration of benefit 9.5 months (95% CI 8.3–25.8)
Trametinib	Trametinib vs standard of care therapies in recurrent LGSOC	GOG-0281 (phase III) (Gershenson et al., 2022b)	Improved PFS (13.0 vs 7.2 months; HR 0.48; *p* < 0.001) and ORR compared with standard of care therapies
Avutometinib + Defactinib	Avutometinib monotherapy vs avutometinib + defactinib in recurrent LGSOC, stratified by *KRAS* mutation status	RAMP 201 (phase II) (Banerjee et al., 2025), RAMP 301 (NCT06072781, phase III) (Grisham et al., 2025)	RAMP 201: combination with ORR 31% (95% CI, 23% to 41%) overall; 44% in *KRAS*m and 17% in *KRAS*wt. RAMP 301 ongoing
Dabrafenib + Trametinib	Combination therapy in treatment-refractory *BRAF*^V600E^-mutated solid tumors	NCI-MATCH (EAY131-H, phase II) (Salama et al., 2020)	ORR of 38% (90% CI 22.9%–54.9%)
Fulvestrant + Abemaciclib	Combination therapy in unresectable, newly diagnosed stage III-IV LGSOC	NCT03531645 (phase II) (Cobb et al., 2022)	CBR 100% with 1/15 patients (7%) with CR and 8/15 (53%) with PR
Ribociclib + Letrozole	Combination therapy in recurrent LGSOC	GOG 3026 (phase II) (Slomovitz et al., 2026)	ORR 30.6% (90% CI 19.9%–43.2%) and CBR 84% (90% CI 72.5%–91.6%)

In the present case, the patient initially received systemic therapy with standard of care carboplatin and paclitaxel, with aromatase inhibitor added after 5 cycles given a partial response on imaging. After completion of 6 cycles, the treating team discussed clinical trial, hormonal therapy and *BRAF*^V600E^-targeted therapy options for persistent disease, though the patient initially declined these due to personal preferences. When she re-presented with widespread metastatic disease, the patient was amenable to BRAF-targeting therapy and to date has shown a marked response to single agent dabrafenib with excellent tolerance. Trametinib has now been added with the goal of delaying resistance and reducing risk of secondary skin cancers. Of note, HER2 immunohistochemistry demonstrated 2+ staining (gastric criteria), a greater extent of expression than most cases reported in the literature, leaving the possibility of treatment with trastuzumab deruxtecan in the future if additional therapeutic options are needed (Němejcová et al., 2025; Badlaeva et al., 2026).

## 4 Conclusions

In summary, SBOT is an ovarian epithelial neoplasm often associated with mutations in genes in the MAPK pathway and can progress to LGSOC. The interval to progression varies widely and, as demonstrated in this case, can occur up to four decades following diagnosis. Improved understanding of the molecular underpinnings of these tumors and advances in molecular testing and targeted therapy have opened doors to more treatment options for LGSOC, which otherwise has limited therapeutic options with variable efficacy. LGSOC lacks consistently effective treatments, and targeted therapies selected by molecular testing may provide a much-needed alternative. Further research to improve the understanding of risk stratification for progression from SBOT to LGSOC may allow for leveraging novel targeted therapies to prevent LGSOC in patients at high risk.

Written informed consent was obtained from the patient for publication of this case report and accompanying images.

## CRediT authorship contribution statement

**Emily A. Towery:** Writing – review & editing, Writing – original draft, Methodology, Formal analysis, Data curation, Conceptualization. **Elizabeth H. Stover:** Writing – review & editing, Investigation, Data curation. **David L. Kolin:** Writing – review & editing, Methodology, Formal analysis, Data curation, Conceptualization. **Rebecca L. Porter:** Writing – review & editing, Supervision, Formal analysis, Data curation, Conceptualization.

## Declaration of competing interest

The authors declare that they have no known competing financial interests or personal relationships that could have appeared to influence the work reported in this paper.
